# Comparison of 3T MR scanners in regional cartilage-thickness analysis in osteoarthritis: a cross-sectional multicenter, multivendor study

**DOI:** 10.1186/ar3174

**Published:** 2010-10-28

**Authors:** Sharon Balamoody, Tomos G Williams, John C Waterton, Michael Bowes, Richard Hodgson, Chris J Taylor, Charles E Hutchinson

**Affiliations:** 1 Biomedical Imaging Institute, The University of Manchester, Oxford Road, Manchester, M13 9PT, UK; 2 Translational sciences, AstraZeneca, Alderley Park, Macclesfield, Cheshire, SK10 4TG, UK; 3 Imorphics Ltd., Kilburn House, Lloyd Street North, Manchester Science Park, Manchester, M15 6SE, UK; 4 NIHR Leeds Musculoskeletal Biomedical Research Unit, Chapel Allerton Hospital, Leeds, LS7 4SA, UK; 5 Clinical Sciences Research Institute, Clinical Sciences Building, University Hospital-Walsgrave Campus, Clifford Bridge Road, Walsgrave, Coventry, CV2 2DX, UK

## Abstract

**Introduction:**

Cartilage thickness from MR images has been identified as a possible biomarker in knee osteoarthritis (OA) research. The ability to acquire MR data at multiple centers by using different vendors' scanners would facilitate patient recruitment and shorten the duration of OA trials. Several vendors manufacture 3T MR scanners, including Siemens, Philips Medical Systems, and GE Healthcare. This study investigates whether quantitative MR assessments of cartilage morphology are comparable between scanners of three different vendors.

**Methods:**

Twelve subjects with symptoms of knee OA and one or more risk factors had their symptomatic knee scanned on each of the three vendor's scanners located in three sites in the UK: Manchester (Philips), York (GE), and Liverpool (Siemens). The NIH OAI study protocol was used for the Siemens scanner, and equivalent protocols were developed for the Philips and GE scanners with vendors' advice. Cartilage was segmented manually from sagittal 3D images. By using recently described techniques for Anatomically Corresponded Regional Analysis of Cartilage (ACRAC), a statistical model was used anatomically to align all the images and to produce detailed maps of mean differences in cartilage-thickness measures between scanners. Measures of cartilage mean thickness were computed in anatomically equivalent regions for each subject and scanner image.

**Results:**

The ranges of mean cartilage-thickness measures for this cohort were similar for all regions and across all scanners. Philips intrascanner root-mean-square coefficients of variation were low in the range from 2.6% to 4.6%. No significant differences were found for thickness measures of the weight-bearing femorotibial regions from the Philips and Siemens images except for the central medial femur compartment (*P *= 0.04). Compared with the other two scanners, the GE scanner provided consistently lower mean thickness measures in the central femoral regions (mean difference, -0.16 mm) and higher measures in the tibial compartments (mean difference, +0.19 mm).

**Conclusions:**

The OAI knee-imaging protocol, developed on the Siemens platform, can be applied to research and trials by using other vendors' 3T scanners giving comparable morphologic results. Accurate sequence optimization, differences in image postprocessing, and extremity coil type are critical factors for interscanner precision of quantitative analysis of cartilage morphology. It is still recommended that longitudinal observations on individuals should be performed on the same scanner and that assessment of intra- and interscanner precision errors is undertaken before commencement of the main study.

## Introduction

Osteoarthritis (OA) is a major and increasing public health problem in the developed world. The present National Institutes of Health Osteoarthritis Initiative (NIH OAI) is a prospective ongoing 9-year study of approximately 5,000 subjects, investigating subjects both with OA and at risk of developing OA to identify measures of OA initiation and progression [1]. Cartilage thickness and volume measurements from MR images are potential morphologic biomarkers for OA that can be assessed from MR images, acquired annually on all subjects in the OAI. Such measures could be used as response biomarkers in the development of therapies for OA, particularly in Phase II clinical trials. All MR images in the OAI are acquired on four Siemens 3.0T MR scanners.

Recent analyses of small initial OAI cohorts suggest cartilage loss in OA to be in the range of 0.5% to 2.0% per annum [2]. Obesity, malalignment, and radiographic OA Kellgren and Lawrence grades 2 to 3 predispose to higher rates of cartilage loss [2-4]. Several factors can influence reproducibility of quantitative measurement of cartilage thickness between groups (for example, age and sex) and within groups (for example, recent weight-bearing activity, diurnal variation [5,6]). Phase II clinical studies of cartilage loss in OA are likely to require a multicenter approach to recruit sufficient numbers of subjects in a reasonable time with adequate statistical power. Most MR scanners are produced by one of three main vendors: Philips (Eindhoven, The Netherlands), Siemens (Erlangen, Germany), and GE Healthcare (Milwaukee, Wisconsin, USA). Multicenter, multivendor clinical studies of OA performed at 3.0T have been published [7]. Although evidence suggests that interscanner differences in cartilage volume in healthy subjects are small [8], little evidence indicates that quantitative analyses of degenerating cartilage on scanners of different vendors are comparable in symptomatic subjects and at higher field strength [9,10].

The study described is a cross-sectional comparison of three 3.0T MR scanners of different manufacturers in the assessment of OA of the knee in a cohort of subjects with symptoms and risk factors for OA. The 3.0T scanners used are manufactured by Philips, Siemens, and GE. The imaging protocols are based on the Siemens NIH OAI protocol, which assesses several features of OA. A quantitative cartilage-morphology analysis is presented. The objectives were to quantify the interscanner precision errors and to assess whether these were significant by comparing with the intrascanner variability (assessed on the Philips scanner).

## Materials and methods

### Subjects

Participants were recruited from the University of Manchester employee base by a universal email sent to all employees. Interested responders were subsequently sent an information sheet and a screening questionnaire. The questionnaire was designed to include contact information, demographics, and assessment of inclusion and exclusion criteria, which were based on those quoted in the NIH OAI protocol [11]. Subjects were required to have one or more symptoms of OA or one or more risk factors for OA or both. OA symptom criteria included pain, stiffness, locking (or requiring analgesia for symptoms) within the last month. Risk-factor criteria were obesity, family history of osteoarthritis, previous knee injury, and frequent knee-bending activity. Responders were excluded if they indicated definite or suspected history of inflammatory arthropathy or had contraindications to MR scanning. Suitable responders were invited for an MR scan. Informed consent was obtained from each participant. Ethical approval for the study was obtained from West Midlands Multi-Centre Research Ethics Committee (06/MRE07/16).

Seventeen subjects (12 men, five women) were initially recruited. Five of these subjects did not complete imaging at all three sites. Two of these five attended but failed to be scanned at one of the sites because of inadequate knee RF coil diameter, and therefore discontinued the study at that point. The other three subjects chose to withdraw before attending all three sites. Data acquired if subjects had not attended one or two of the sites was excluded from analysis, and therefore, the data for the 12 subjects who had been scanned at all three sites were analyzed. Demographics for this cohort *n *= 12 as follows: nine men, three women,, aged 49.3 ± 10.0, 32 to 58 years (mean ± standard deviation, range) and BMI 28.3 ± 6.2, 22.1 to 44.2 kg/m^2^. Five of 12 subjects had knee pain on activity or rest or both for at least 7 days in the last month at the time of completing the questionnaire; the other seven subjects experienced pain less frequently. Three subjects previously had received a diagnosis of OA.

Although radiographic data were not available to demonstrate the presence of definite OA features, such as joint-space loss and osteophytes, for volunteer selection, as in the NIH study, semiquantitative knee scoring by using Whole-Organ Magnetic Resonance Score (WORMS) [12] was performed by a single observer (SB) on the acquired MR data. The WORMS involves dividing the total knee cartilage into 14 subregions (4 points for the patellofemoral (PF) joint; 5, for medial tibiofemoral (MTF) joint; and 5 for lateral tibiofemoral (LTF) joint). The cartilage in each subregion is scored from 0 to 6; 0 = normal cartilage; 6 = diffuse full-thickness cartilage loss. Then the relevant subregions are summed to obtain compartmental scores. With WORMS, maximal compartmental values obtainable would be 30, 30, and 12 for the MTF, LTF, and PF joints, respectively.

### The scanners

The Siemens sequences were based on the NIH OAI protocol [11], whereas optimized protocols were obtained for the Philips and GE scanners. This was done with the help of clinical scientists specializing in musculoskeletal MR from Philips (Netherlands) and GE (UK). A summary of Siemens 3D DESS-we, and equivalent sequence parameters on the other scanners is given in Table 1. A notable difference is apparent with the GE sequence, which uses fat saturation in place of water-excitation and also has a lower acquired resolution. Philips uses a higher TE/TR compared with the other two scanners (9/20 ms versus 4/16 ms).

**Table 1:** Summary of sequence parameters for the Siemens OAI protocol and optimized equivalent protocols for the GE and Philips 3.0T scanners

Vendor	Siemens	Philips	GE
Sequence	3D DESS	3DWATSf	3D GE
Fat saturation/water excitation (WE)	WE	WE	Fat sat
FOV (mm)	140	140	140
Number of slices	160	120	210
Slice thickness (mm)	0.7	0.7	1
Slice gap (mm)	0	0	-0.5
Flip angle (deg)	25	25	25
TE (ms)	4.7	9.2	4.9
TR (ms)	16.3	20	16.8
X-resolution (mm)	0.37	0.36	0.55
Y-resolution (mm)	0.46	0.48	0.62
Scan time	10 min 35 s	3 min 51 s	6 min 22 s

D.ESS, dual-echo steady state; GE, gradient echo; WATSf, water selective (fluid).

The three 3.0T MR scanners were located as follows: Philips Achieva (Manchester, UK), Siemens Trio (MARIARC, Liverpool, UK), and GE Signa (YNIC, York, UK). The Siemens and GE coils used were quadrature circularly polarized transmit-receive and single-channel transmit-receive multipurpose HD knee/foot coil, respectively. An eight-channel SENSE phased-array receive coil was used at the Philips site. The SENSE function on the Philips coil was implemented for certain sequences only.

### Data acquisition

Each subject was designated to have the same knee examined on each of the three different vendors, and, at the Philips 3.0T scanner, an additional examination at the same visit. Before scanning at each center, each subject underwent a minimum rest period of non-weight bearing of at least 30 minutes to minimize variability in cartilage thickness between scans due to recent weight-bearing activity. It was not possible totally to control for within-subject diurnal variation in cartilage thickness between scans because of constraints on scanner and subject availability. Scan times for the OAI protocol (which comprised five acquisition sequences imaging the knee) at the Siemens, Philips, and GE scanners were 37 minutes 41 seconds, 39 minutes 9 seconds, and 41 minutes, respectively. At the Philips site, the sagittal 3D WATSf was acquired twice within the same session to assess intrascanner precision. After the first acquisition, the subject was removed from the magnet bore and allowed to sit on the scan table without weight bearing for approximately 10 to 15 minutes and repositioned for the second acquisition.

Efforts were made to ensure similar orientation of slices between scanners as follows. The planning for the first sagittal and coronal sequences was applied to subsequent sequences for each scan at each center. After the first of the three scans, the overlays of the scout views for sagittal and coronal images were saved and used for comparison for planning subsequent scans of the same subject on the other two scanners. Images at each of the sites were labeled with a unique study number randomized independently at each site so that observers would be blind to subject identity for each of the scanners. Blinding to scanner was not performed, as scanners could be easily identified from the images by their appearance.

### Quality assurance

The routine quality assurance (QA) at each scanner site was recorded as follows. At the GE site, magnet SNR checks were performed on alternate days. No other documented quality control was available.

At the Siemens site, monthly/2-monthly QA reports were documented, including SNR, gradient sensitivity, and fat/water saturation checks. Some of the QA for the Siemens site was performed remotely.

At the Philips site, daily checks were performed by the radiographers. A dedicated employee responsible for scanner QA was available to perform checks by using phantoms investigating geometric distortion, SNR, RF uniformity, and slice profile. Most of the tests were stable. Regarding geometric distortion, slight drift was observed in the craniocaudal direction (-0.1% to 0.2%) over the time course of the study. RF coil SNR checks were performed only occasionally at all sites.

### Cartilage segmentation

In total, 48 scans were included in the cartilage morphology analysis: 12 subjects × four scans (three sites + extra 3D WATSf dataset acquired at Philips scanner). The cartilage was manually segmented from the sagittal 3D sequences by a single observer (author 1) blinded to subject identity, by using the Endpoint segmentation software (Imorphics, Manchester, UK), which implements a livewire algorithm [13]. Quality control of all segmentations was conducted by an experienced consultant musculoskeletal radiologist (last author). Closed-surface representations of the cartilage sheets were generated from the segmentations to enable volume and thickness to be measured in 3D.

### Defining a common frame of reference: bone reference surfaces

**Figure 1:**
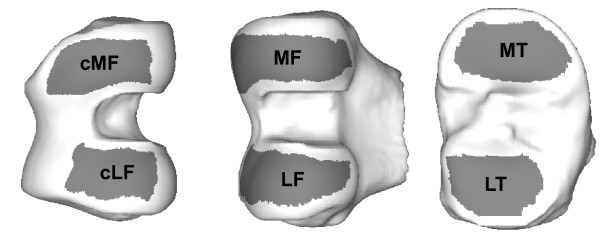
**Anatomic regions of interest illustrated on the mean bone shape**. All regions are trimmed to exclude the periphery of the cartilage. Two regions are defined within both femoral condyles: the full region extending to the posterior of the joint; and central (c), restricted to the main load-bearing regions.

To compare measurements of cartilage thickness from images of the same subject taken with different scanners, and to enable aggregation of measures over the study cohort, an anatomic frame of reference was defined in each image by using recently described techniques for Anatomically Corresponded Regional Analysis of Cartilage (ACRAC) [14]. The exosteal bone surface was chosen as a basis for the frame of reference because of the relative ease of its identification in the images and consistent topology across subjects. Six regions were defined on this surface; these were used for the analysis (see Figure 1). Statistical models were used as a means of defining the bone reference surface in each image [15]. The models provide a mean representation of all the training-set images and a warp from the mean image to each example image. When presented with new MR images, which were not part of the original training set, statistical models can also automatically find the best warp field from the mean image.

A statistical model was built from the DESS-we images of both knees fromthe 160 participants initially released by the OAI as group 0.B.1, which included a single time point only and both knees of participants [1]. The exosteal bone boundary was segmented by a consultant musculoskeletal radiologist (last author) in the mean model image, and a surface representation was generated. The warp fields from the mean image to the study images were found by the model and used to propagate the bone reference surface, thus providing anatomically coherent bone segmentations for each scan.

### Cartilage morphology measures

The bone reference surfaces provided a dense set of points with mean separation of 0.6 mm on the distal femur and proximal tibia. Cartilage thickness was measured above each point in each image as the distance between the points of intersection of the 3D line, normal to the bone reference surface, with the inner and outer cartilage surfaces. The result is a cartilage-thickness measurement associated with each measurement point, which was displayed as a color-coded map of cartilage thickness on each reference bone surface.

From the individual cartilage-thickness maps, summary measures of cartilage morphology measures within anatomically consistent regions were computed to enable us to perform statistical analysis of the differences. From the bone reference surface, regions of interest, consistent with those proposed by Eckstein *et al*. [16] were delineated on the mean distal femoral and proximal tibial bone surfaces. Mean cartilage thickness over total bone area (ThCtAB, as defined by Eckstein *et al*. [16]), was computed for all regions as the average of the point-wise thickness measurements weighted by the area surrounding each measurement point. These regions by definition do not exclude any areas denuded of cartilage. Peripheral cartilage is more prone to segmentation error due to partial volume effect, as the surface curves into the image plane. In common with other authors [17,18], we remove the outer edges of cartilage coverage by defining trimmed regions for each joint. Figure 1 illustrates the six regions of interest, trimming boundary, and identifying labels, as shown on the mean bone surfaces. This technique has been described in detail by Williams *et al*. [19].

### Population and difference maps

The correspondence of the cartilage-thickness maps between scan sessions and across subjects enables us to aggregate the individual subjects' thickness maps to a mean cohort-thickness map for each scanner. Individual maps could be constructed to demonstrate the point-wise differences in thickness between scanner pairs, and these are also combined to form mean cartilage-thickness difference maps for the cohort. These population-difference maps were displayed on the mean bone shape, from which more-localized regional measures could be performed.

### Statistical analysis

The study provided data for the reproducibility analysis of regional cartilage mean thickness between scanners (interscanner) and within the Phillips scanner (intrascanner). Image data from the first scan of the two taken at the Philips site were used only for the interscanner analysis. Intra- and interscanner precision was evaluated with Bland-Altman analysis, paired *t *tests, and coefficient of variation (COV), aggregated as the root-mean-square over all subjects for scanners pairs and expressed as a percentage [20]. Bland-Altman analysis provides the 'bias' (mean difference) and its limits of agreement between any pairs of measurements [21].

## Results

All data were acquired within a 4½-month period. The duration between first and last scans for a single subject was 74 ± 35 days; range, 13 to 123 days. Results of the semiquantitative cartilage scoring by WORMS [12] demonstrated a large range of changes within the cohort. Mean cartilage scores for the MTF, LTF, and PF compartments for the cohort were 5.7 ± 5.9, 3.6 ± 4.7, and 7.0 ± 5.1, respectively (Philips scanner) [22]. Subjects demonstrated a broad range of OA pathology, from mild cartilage abnormalities to full-thickness cartilage loss and subchondral changes.

### Intrascanner precision

The Philips intrascanner COVs based on the two scans were in the range 2.6% to 4.6%. Low variability is also depicted by the Bland-Altman analysis, which produced mean differences of magnitude ≤ 0.05 mm and tight 95% limits of agreement of magnitude < 0.35 mm and no significant differences between the two scans (paired *t *test, *P *> 0.05) for all regions except the MF region, for which the difference was small at 0.05 mm (Table 2).

**Table 2:** Results of Philips intrascanner and vendor interscanner comparisons

Scanner pair	Region	RMS COV	Mean difference/bias (mm)	95% limits of agreement (mm)		Paired *t *test
				Lower bound	Upper bound	*P *value
Philips first to second scan(intrascanner)	cLF	2.6%	0.00	-0.19	0.20	0.95
	cMF	3.2%	0.03	-0.14	0.20	0.23
	LT	4.1%	-0.05	-0.34	0.25	0.30
	MT	4.6%	0.00	-0.30	0.30	0.98
	LF	3.6%	0.00	-0.23	0.23	0.98
	MF	2.8%	0.05	-0.08	0.18	0.02^a^
Philips to Siemens	cLF	4.1%	0.06	-0.13	0.25	0.10
	cMF	5.4%	0.05	-0.19	0.30	0.04^a^
	LT	4.0%	0.06	-0.10	0.22	0.75
	MT	5.6%	0.05	-0.33	0.43	0.30
	LF	4.9%	0.13	-0.04	0.30	0.00^a^
	MF	5.8%	0.08	-0.12	0.29	0.00^a^
Siemens to GE	cLF	5.1%	0.12	-0.11	0.36	0.00^a^
	cMF	5.6%	0.13	-0.06	0.31	0.00^a^
	LT	7.7%	-0.24	-0.55	0.06	0.00^a^
	MT	7.7%	-0.17	-0.51	0.18	0.01^a^
	LF	4.2%	0.04	-0.20	0.27	0.28
	MF	5.2%	0.01	-0.25	0.26	0.85
Philips to GE	cLF	6.6%	0.18	-0.03	0.39	0.00^a^
	cMF	9.6%	0.21	-0.03	0.46	0.00^a^
	LT	8.1%	-0.23	-0.60	0.13	0.00^a^
	MT	5.4%	-0.12	-0.34	0.11	0.00^a^
	LF	7.2%	0.17	-0.14	0.47	0.00^a^
	MF	7.3%	0.14	-0.14	0.42	0.00^a^

^a^*P *< 0.05 (between-scanner differences are significant). The Bland-Altman plots (not shown) showed no relations between mean and difference of per-subject measures of regional cartilage thickness. cLF, central lateral femur; cMF, central medial femur; LF, lateral femur; LT, lateral tibia; MF, medial femur; MT, medial tibia; RMS COV, root-mean-square coefficient of variation.

### Interscanner precision

For the three scanner pairs, interscanner cartilage volume COVs were on average 2.3% higher than thickness values for the six regions. Overall, the comparison of the Philips and Siemens scanners gave values similar to those of the Philips intrascanner. The Philips 3D WATSf mean cartilage-thickness measures were ≤ 0.06 mm thicker across all four main weight-bearing regions (cMF, cLF, LT, and MT) compared with the 3D DESS (Siemens). These small differences were nonsignificant (paired *t *test, *P *> 0.05), except for the central medial femur, the result of which can mostly be attributed to an outlier measurement of abnormally thin cartilage in the Siemens scan of one of the subjects.

**Figure 2:**
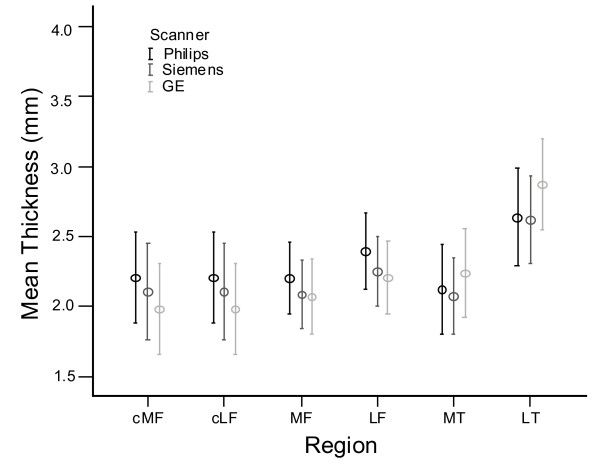
**Mean and 95% confidence interval (CI) for mean thickness values for all regions and all scanners**.

More pronounced differences in cartilage-thickness measurements were found on the GE scanner compared with the other two vendors' scanners, as is evident in Figure 2. Measures of the mean thickness in the central femur regions were consistently lower on the GE scanner (average bias, 0.13 mm over the four femoral regions and both scanner pairs) and consistently higher in the tibial compartments (average bias, 0.19 mm). Significant interscanner differences were found for several regions, especially when compared with the Philips data (*P *< 0.05).

### Thickness-difference maps

**Figure 3:**
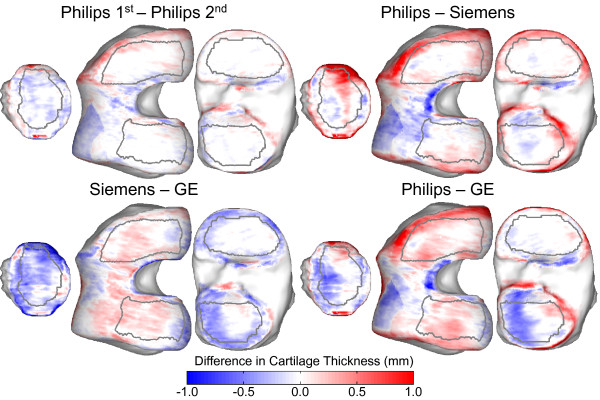
**Mean cartilage thickness interscanner and intrascanner difference maps shown on the mean bone surfaces**. Differences are shown by using the same blue-to-red color scale for all comparisons, with red indicating thicker cartilage in the first compared with the second scanner.

The spatial distribution of differences between scanners is visualized on the mean cartilage-difference thickness maps in Figure 3. The differences are shown by using a consistent red-to-blue scale for all four comparison plots. In general, the largest variability is exhibited at the periphery of the cartilage coverage. The paler-color map for the Philips intrascanner differences reflects the lower variability compared with the interscanner maps. The Philips and Siemens scans showed higher values for femoral cartilage thickness compared with GE, whereas this pattern was reversed in the tibia.

## Discussion

The current interest in multicenter trials using MR-imaging biomarkers to measure OA progression [7] requires investigation and optimization of interscanner precision errors. Multicenter trials using 3.0T scanners may require using scanners of different vendors, and therefore acquisition sequences and hardware may differ between sites. This study is the first to compare repeated measures of cartilage morphology from 3.0T MR scanners of three different vendors on symptomatic subjects.

Intrascanner cartilage mean thickness coefficients of variation on the Philips scanner in this study are in the range 2.6% to 4.6%, which is in line with other intrascanner analyses in OA [23,24]. Interscanner variability between the Philips and Siemens scanners demonstrated small systematic differences in regional cartilage-thickness measurements that were comparable with the intrascanner precision errors. The results compare favorably with those of the previous multivendor study by Morgan *et al*. [8], which measured knee-cartilage volume in healthy subjects at 1.0 to 1.5T, where interscanner volume COVs were in the range 9.0% to 18.7%. This can be explained by the increased SNR, which allowed resolution to be almost doubled in each plane, reducing partial-volume effects. In our study, intrascanner cartilage-volume COVs produced very similar values to the thickness COVs, whereas interscanner volume COVs were on average 2.3% higher for the given regions. Volume COVs are a function of variability of both thickness and surface area, thus providing limited information on sources of variability in precision errors and therefore not reported in detail here.

The higher interscanner variability measured between Philips and GE can be explained by both hardware and sequence differences. Difficulties were encountered in mimicking the DESS sequence on the GE platform. Increased image noise and differences in sequences (water-excitation on Philips versus fat-saturation on GE) may have an effect on the cartilage boundaries. Comparisons of these sequences have demonstrated apparently thicker cartilage in the water-excitation images, although the differences were in the range of *in vivo *reproducibility [24]. Although image resolutions were similar, subjectively, the GE images appeared more noisy. Poststudy examination of the images revealed that the cartilage-to-bone boundary was subjectively less pronounced on the GE scanner compared with the Philips scanner and that, when comparing images of the same subjects, in some cases, the femoral cartilage appeared thinner. Partial-volume errors are consequently likely to have been greater. With respect to segmentation of the sagittal 3D sequences; the Philips images were the easiest to segment, believed to be due to postprocessing, which eliminated background noise and accentuated the cartilage edges, aiding the livewire algorithm in segmentation. The Siemens and GE images appeared noisier, although the Siemens DESS sequence provided superior fluid-to-soft tissue contrast at the cartilage surface. Formal assessment of image SNR could not be performed because of the postprocessing of the Philips images. These factors also may account for the underestimation of femoral-cartilage thickness on the GE scanner due to more difficulties encountered delineating the cartilage-to-bone boundary.

A significant variation was found in the QA performed at each site, with formal slice-thickness profile and geometric distortion QA being available at the Philips site only. It is possible that variations in the slice-thickness profile between scanners may have contributed to the interscanner variation because of differences in partial-volume effects. These errors are considered to be most frequent at sites of greater surface curvature at the edges of the cartilage plates, which would have been reduced by the trimming of the segmented cartilage surfaces in the image analysis.

Multivendor studies investigating cartilage morphology have been performed previously by using MR scanners at 3.0T and lower field strengths [8,9]. Morgan *et al*. [8] detected small systematic interscanner differences in quantitative measurements of cartilage volume. Kornaat *et al*. [9] performed a study with two 3.0T scanners manufactured by Philips and GE. The principal difference of our study is that the subjects are symptomatic. Accuracy and precision of cartilage measurements in thinned cartilage is more challenging because of factors such as partial-volume averaging and altered signal and structure in pathologic tissues. Potential factors affecting variability between scanner vendors include differences in hardware, pulse sequences, and so on. At 3.0T, factors such as B_1 _and B_0 _inhomogeneity play a greater role in interscanner variability compared with 1.5T [25]. It is important to emphasize that our study compared three specific scanners, and the results cannot necessarily be generalized to all scanners of a particular vendor. We aimed to investigate the likely errors in a multivendor study, not to compare the performances of different vendors' products in general.

Interscanner differences in knee-cartilage thicknesses between Philips and GE 3.0T scanners have been measured by others in healthy subjects [9], where a mean difference of 0.19 mm (up to a maximum of 0.88 mm but not statistically significant) in the femoral-cartilage thickness was reported. Similar to this study, the Philips scanner used had an eight-channel phased-array knee RF coil, and the GE scanner had a quadrature transmit-receive coil. Hardware differences are inevitably encountered in other multicenter studies, as often only one RF coil type is available, as was the case in this study.

Minimizing precision errors means that smaller differences can be detected in a group with a given number of subjects. What represents a clinically significant difference in cartilage thickness in OA is still unclear and, we hope, will be answered by the results of the NIH OAI study. This study presents a method intended for monitoring changes in groups of subjects with OA. Our method incorporates a bone-modeling technique that aims to facilitate image segmentation and analysis by achieving anatomic correspondence between images. The model was devised by using data from a training set of images from Siemens scanners. We have shown that this can be successfully applied to similar image data from scanners of different vendors.

## Conclusions

The study demonstrates that the Siemens NIH OAI protocol can be optimized for the Philips and GE 3T scanners to achieve images of similar resolution and contrast. However, in quantitative cartilage-thickness analysis, significant interscanner differences may occur in some cases. These may be due to a combination of differences in extremity RF coil, challenges in sequence optimization, and image postprocessing.

Overall, imaging at 3.0T improves intra- and interscanner precision errors in comparison with imaging at lower field strengths. This study supports the use of 3.0T scanners from different manufacturers in clinical trials of OA involving quantitative MR cartilage morphology after investigation of interscanner precision errors. It is still recommended, however, that longitudinal observations on individuals be performed on the same scanner.

## Abbreviations

COV: coefficient of variation; DESS-we: double echo steady state-water excitation; Fat Sat.: fat saturation; FOV: field of view; LTF: lateral tibiofemoral; MTF: medial tibiofemoral; NIH: National Institutes of Health; OA: osteoarthritis; OAI: osteoarthritis initiative; PF: patellofemoral; SENSE: sensitivity encoding; SNR: signal-to-noise ratio; TE: echo time; TI: inversion time; WATSf: water selective (fluid); WE: water excitation; WORMS: whole-organ magnetic resonance score.

## Competing interests

CJT and MB declare the following financial competing interests: CJT is currently Principal Investigator on a research council grant that involves collaboration with Imorphics, Ltd., continuation of which is dependent on a significant contribution in kind from the company; and MB is an employee of the company. CJT and MB personally hold shares in Imorphics, Ltd., which is a University spin-out company. Imorphics provides products and services for the quantitative analysis of medical images for clinical trials and holds the commercial exploitation rights for the work described in the article.

SB, CEH, JCW, RH, and TGW declare that they have no competing interests.

## Authors' contributions

SB is Principal Investigator, and CEH is Chief Investigator for this study. SB, CEH, JCW, and TGW contributed to the conception and design of the study and in the interpretation and discussion in the manuscript. TGW, MB, and CJT contributed to quantitative image analysis. RH contributed to image sequence optimization and data acquisition. All authors read and approved the final manuscript.
